# Molecular imaging research in atherosclerosis: A 23-year scientometric and visual analysis

**DOI:** 10.3389/fbioe.2023.1152067

**Published:** 2023-04-13

**Authors:** Juhong Pan, Yueying Chen, Yugang Hu, Hao Wang, Wenwei Chen, Qing Zhou

**Affiliations:** Department of Ultrasound Imaging, Renmin Hospital of Wuhan University, Wuhan, China

**Keywords:** molecular imaging, atherosclerosis, scientometric, VOSviewer, citespace 6

## Abstract

**Background:** Cardiovascular and cerebrovascular diseases are major global health problems, and the main cause is atherosclerosis. Recently, molecular imaging has been widely employed in the diagnosis and therapeutic applications of a variety of diseases, including atherosclerosis. Substantive facts have announced that molecular imaging has broad prospects in the early diagnosis and targeted treatment of atherosclerosis.

**Objective:** We conducted a scientometric analysis of the scientific publications over the past 23 years on molecular imaging research in atherosclerosis, so as to identify the key progress, hotspots, and emerging trends.

**Methods:** Original research and reviews regarding molecular imaging in atherosclerosis were retrieved from the Web of Science Core Collection database. Microsoft Excel 2021 was used to analyze the main findings. CiteSpace, VOSviewer, and a scientometric online platform were used to perform visualization analysis of the co-citation of journals and references, co-occurrence of keywords, and collaboration between countries/regions, institutions, and authors.

**Results:** A total of 1755 publications were finally included, which were published by 795 authors in 443 institutions from 59 countries/regions. The United States was the top country in terms of the number and centrality of publications in this domain, with 810 papers and a centrality of 0.38, and Harvard University published the largest number of articles (182). Fayad, ZA was the most productive author, with 73 papers, while LIBBY P had the most co-citations (493). *CIRCULATION* was the top co-cited journal with a frequency of 1,411, followed by *ARTERIOSCL THROM VAS* (1,128). The co-citation references analysis identified eight clusters with a well-structured network (Q = 0.6439) and highly convincing clustering (S = 0.8865). All the studies calculated by keyword co-occurrence were divided into five clusters: “nanoparticle,” “magnetic resonance imaging,” “inflammation,” “positron emission tomography,” and “ultrasonography”. Hot topics mainly focused on cardiovascular disease, contrast media, macrophage, vulnerable plaque, and microbubbles. Sodium fluoride ⁃PET, targeted drug delivery, OCT, photoacoustic imaging, ROS, and oxidative stress were identified as the potential trends.

**Conclusion:** Molecular imaging research in atherosclerosis has attracted extensive attention in academia, while the challenges of clinical transformation faced in this field have been described in this review. The findings of the present research can inform funding agencies and researchers toward future directions.

## Introduction

Cardio-cerebrovascular and peripheral vascular diseases as the leading causes of death and substantial loss of health have remained a major global health concern (Mensah et al., 2019; Roth et al., 2020; Vaduganathan et al., 2022), and atherosclerosis (AS) is the main pathological basis contributing to such diseases (Al Rifai et al., 2022; Mohanta et al., 2022). AS is a chronic inflammatory disease that starts in childhood and involves the blood vessel walls of multiple systems (Schipper and de Ferranti, 2022). Moreover, AS is insidious for a long period until the plaque ruptures and thrombosis forms, which leads to heart attack, and stroke (Borne et al., 2017; Lv et al., 2022). Although imaging detections of cardio-cerebrovascular diseases (Wiegers et al., 2020; Williams et al., 2022) have been increasingly developed, the conventional imaging methods focused on displaying anatomy and structure; for instance, ultrasound research to assess the intima-media thickness (IMT) of the common carotid and plaque size and computed tomography (CT) evaluation of coronary artery calcification (Colantonio et al., 2018), cannot provide information on the underlying pathophysiological processes related to the early stage and complications of AS. How to effectively use non-invasive diagnostic procedures to monitor the occurrence and progression of AS has become an urgent problem.

Molecular imaging was first proposed in 1999 by Professor Weissleder from Harvard University at the International Imaging Conference held in Mississippi. Just as the name implies, molecular imaging can reflect changes at the molecular and subcellular level *in vivo* and conduct qualitative and quantitative research on biological behaviors (Duan et al., 2022), which has been widely employed in the diagnosis and therapeutic applications of a variety of diseases (Hughes et al., 2022; Miao et al., 2022), including atherosclerosis. Characteristics of vulnerable plaque, such as angiogenesis, inflammation, necrotic core, intraplaque hemorrhage, thin fibrous cap, and microcalcification can be assayed (Bala and Cosyns, 2014; Dweck et al., 2016).

With the rapid development of nanotechnology, bioengineering, genomics, and medical imaging, large numbers of publications (Douma et al., 2009; Ahmed et al., 2020; Shentu et al., 2021) have announced that molecular imaging has broad prospects in the precise diagnosis and treatment of atherosclerosis. However, as far as we know, there are few research works on scientometric analysis regarding this quickly developing field.

Scientometrics is an application-oriented discipline that describes the process of scientific development, reveals the internal mechanism of scientific evolution, and predicts the trend of scientific development. It takes quantitative analysis as the main way to reflect the scientific activities of a certain domain (Lackner et al., 2021; Zhou et al., 2022). Meanwhile, there have been several kinds of software developed to help scholars manage data mining and knowledge mapping such as CiteSpace (Fan et al., 2020), VOSviewer (van Eck and Waltman, 2010), HistCite (Li et al., 2022a), and so on. Among them, the former two are the most widely used in clinical medical fields such as respiratory diseases (Yang et al., 2022b), cardiovascular diseases (Chen et al., 2020), and nanomedical fields (Zhu et al., 2020; Cheng et al., 2022; Zhao et al., 2022).

In this study, we conducted a scientometric analysis based on state-of-the-art methods with the aim to evaluate how molecular imaging research in AS has evolved from 2000 to 2022 in terms of hotspots and emerging trends. We then describe the challenges of clinical transformation faced in this field, which may provide a macroscopic view for new researchers and also be beneficial for funding agencies to grasp future research hot issues.

## Methods

### Data source and acquisition

The Science Citation Index Expanded (SCIE) was selected as the data source. Created as SCI in 1964 and as one of the most core databases in the Web of Science Core Collection (WOSCC), SCIE is the world’s most influential index database of multi-disciplinary academic literature abstracts, which now indexes over 9,500 of the most impactful journals across 182 scientific disciplines. More than 61 million records date back from 1900 to the present (Last Updated: 30 March 2023), and it has been recognized as the most authoritative literature retrieval tool by the global academia in science and technology.

**FIGURE 1:**
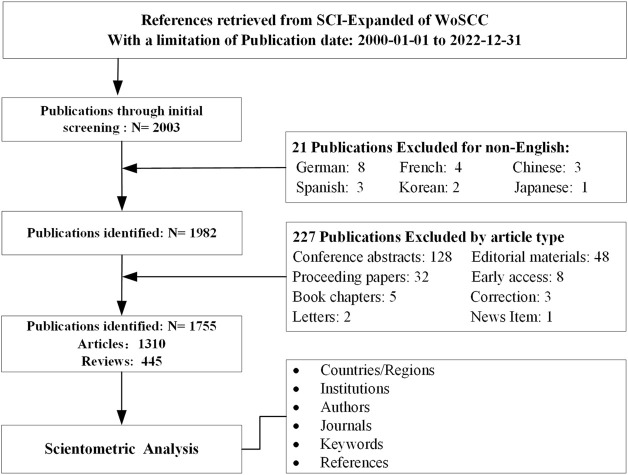
Flowchart of the study strategy.

In order to avoid database update deviation, two researchers of our team searched the literature on molecular imaging in AS simultaneously and completed the retrieval on a single day (24 March 2023). Search session Queries: #1: ((((((((TS = (Atherosclero*)) OR TS = (Atherogen*)) OR TS = (Arteriosclero*)) OR TS = (Atheroma*)) OR TS = (Atherosis)) OR TS = (Scleratheroma)) OR TS = (Fibroatheroma)) OR TS = (“Arterial Fatty Streak*ˮ)) OR TS = (“Arterial lipoidosis”) Timespan: 01-01-2000 to 31-12-2022; #2: (((((((((((((TS = (“Molecul* imaging”)) OR TS = (*target* contrast media)) OR TS = (*target* contrast agent*)) OR TS = (*target* contrast Material*)) OR TS = (*target* imaging probe*)) OR TS = (Nanoagent* imaging)) OR TS = (Nanoprobe* imaging)) OR TS = (Nanoparticle* imaging)) OR TS = (Nanomaterial* imaging)) OR TS = (Nanomedic* imaging)) OR TS = (Nanobubble* target* imaging)) OR TS = (Microbubble* target* imaging)) OR TS = (Nanodroplet* target* imaging)) Timespan: 01-01-2000 to 31-12-2022; Then search: #1 AND #2 and English (Languages) and Article or Review Article (Document Types) and Proceeding Paper or Early Access or Book Chapters (Exclude—Document Types). Subsequently, full records and cited references of the literature were exported and stored in plain text format. A detailed study strategy is shown in Figure 1.

### Data analysis and visualization

The data given in the aforementioned were analyzed independently by two researchers in order to guarantee accuracy and repeatability.

An online scientometric platform (http://bibliometric.com/) that won the third prize in the 2013 National Science Library of the Chinese Academy of Sciences' Scientific Research Education Open Information Innovation Application Contest' was applied to visualize the collaboration and publication analyses of countries/regions.

CiteSpace (Version 6.2.R2 Basic, downloaded from https://citespace.podia.com, last updated on 26 March 2023) is a popular citation visualization software (Synnestvedt et al., 2005; Novo et al., 2013; Shen et al., 2022) which was developed based on Java by Professor Chaomei Chen from the School of Information Science and Technology of Drexel University and WISE Laboratory of Dalian University of Technology, and we know that CiteSpace does a good job in literature timeline analysis and keywords burst. In this study, it was used to perform different kinds of visualization analysis, including co-authorship of nations, authors and organizations, co-citation of authors, papers, and journals, in addition to references timeline analysis and keywords burst, with the purpose of revealing the knowledge structure and acquiring insights into the application of molecular imaging research in AS based on massive data. Of note, when it came to the clustering function, the scores of Modularity Q and Mean Silhouette had a significant impact on the overall network. When Q >0.3, it reveals a well-structured network. When S >0.5, it indicates that the cluster is logical, and when S is more than 0.7, it shows that the cluster is effective and credible (Schneider, 2004). Centrality was a parameter that was used to measure the bridging role of nodes in the overall network. If centrality >0.1 represents high betweenness centrality of the nodes, the so-called critical nodes are depicted in purple rings in bibliometric maps.

VOSviewer (version 1.6.18) is another widespread scientific knowledge mapping software designed by van Eck and Waltman from Leiden University (van Eck and Waltman, 2010), with advantages of easy operation and quick running, as well as better clustering analysis and visualizing the bibliometric network (van Eck and Waltman, 2017; Yang et al., 2022a; Vittori et al., 2022). In the current research, it was adopted to conduct co-authorship of organizations and co-occurrence of author-keyword analyses.

Microsoft Excel 2021 was employed to analyze descriptive statistics containing the most productive or top-cited authors, institutions, papers, and journals. In addition, the 2021 journal impact factor (IF), Journal Citation Reports (JCR), and H-index were gotten from WOSCC. H-index was a citation index of famous papers proposed by John Hirsch, which is commonly used nowadays to estimate scientists’ academic achievements (Ioannidis et al., 2019).

## Results

### Trends of overall publications and citations

**FIGURE 2:**
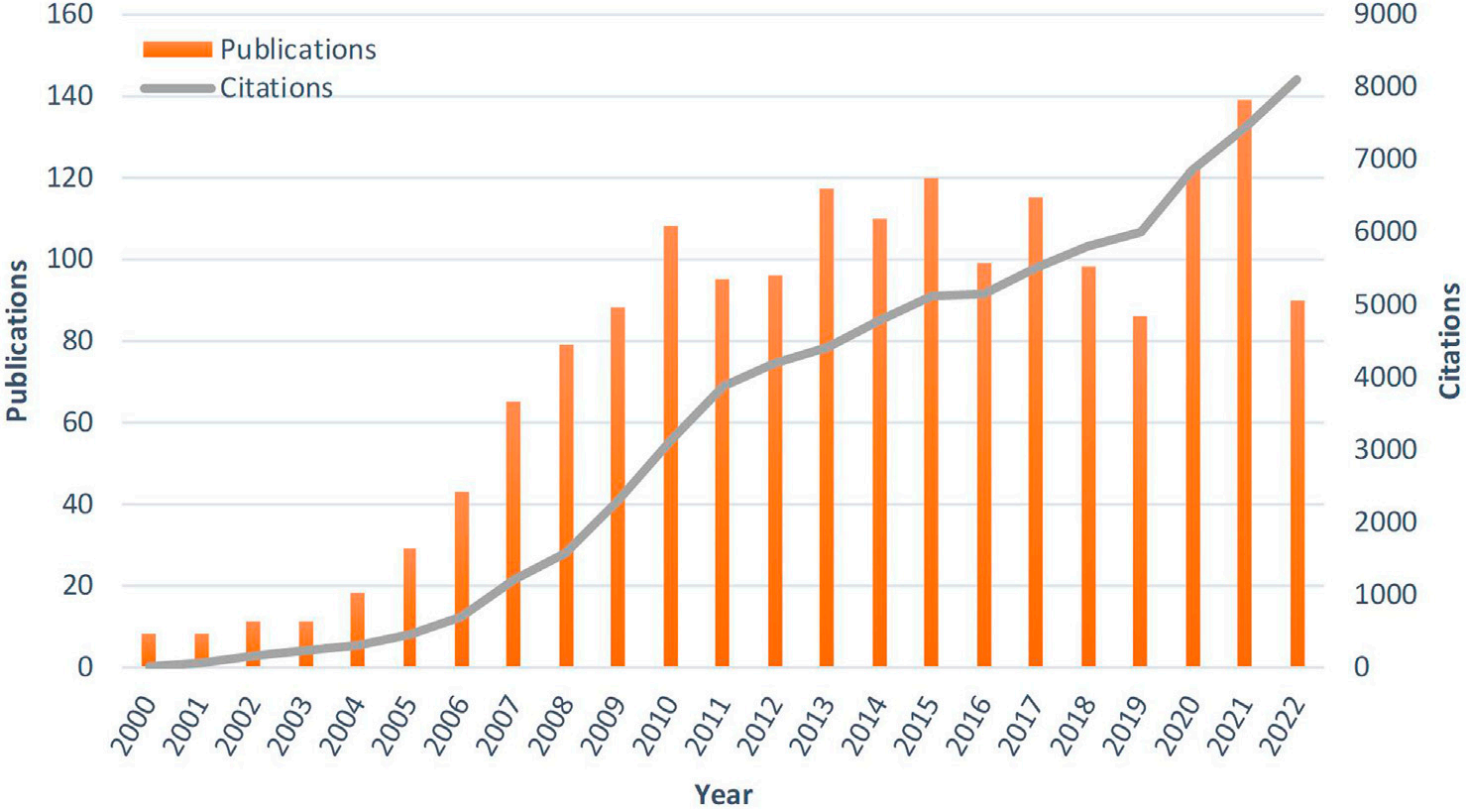
Trends of overall publications and citations on molecular imaging research in AS from 2000 to 2022.

From 2000 to 2022, a total of 1755 publications were finally included, containing 1,310 articles and 445 reviews (Figure 1). It can be seen from Figure 2 that the global trend of molecular imaging research in AS can be divided into two stages: 1) 2000 to 2010 was a rapid growth period, and the publications had been increasing explosively from 8 (2000) to 108 (2010). 2) From 2011 to 2022, molecular imaging research in AS underwent a stable development period accompanied by some fluctuations. Remarkably, the annual outputs stayed on a high level, with an average of 107 articles per year, and the number of publications in this period accounted for 73.3% (1,287/1,755) of the total publications. Furthermore, all the scientific literature had been highly cited, with a total cited frequency of 77,233 times, and with an average of 44 times for each paper. Citations represented a similar overall developing trend.

### Analysis of co-authorship network of countries/regions

**FIGURE 3:**
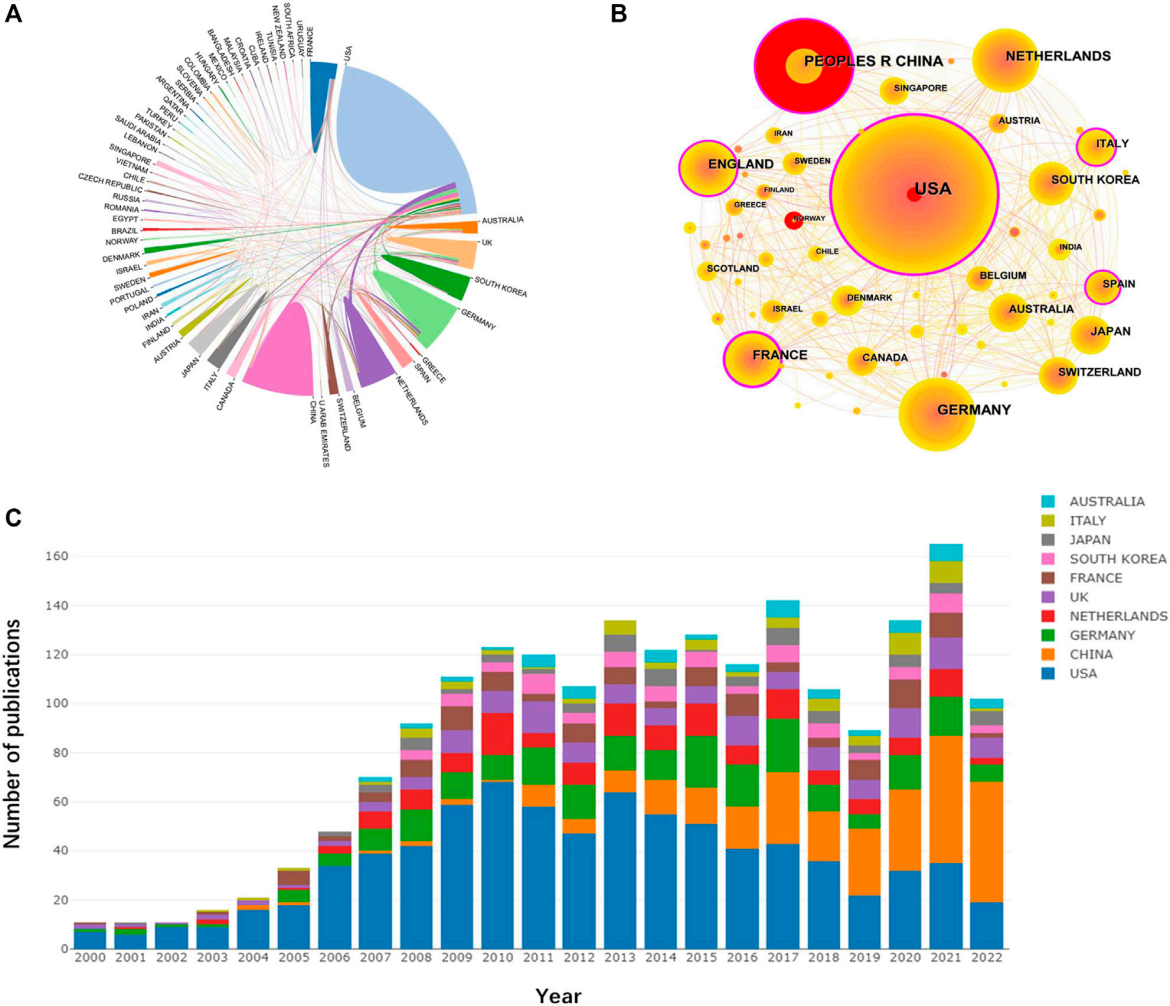
**(A)** The network of collaborating countries generated from an online bibliometric platform. The thickness of the line between countries reflects the strength of the cooperation relationship. **(B)** The network of collaborating countries/regions generated by Citespace. Highly central nodes are the outermost purple ring. Countries presenting the highest centrality are at the center of the network, which is considered the pivotal point in the research field. The thickness of the outermost red ring indicates the burst level. **(C)** The trend of the annual publications in the top 10 countries/regions from 2000 to 2022.

Related articles published in this field were from 59 countries/regions. As shown in Figures 3A, B, the United States was leading the research on molecular imaging research in AS and had collaborated closely with Germany, Netherlands, and China. Table 1 shows that the top five productive countries include the United States (810), China (283), Germany (227), Netherlands (151), and England (130), accounting for 91.2% (1,601/1755) of the total publications. Among them, China started research in this field later than others chronologically from 2004 (Table 1) but had publications burst in recent years marked by the outermost red thick ring of the node (Figure 3B). The countries/regions colored with purple in Figure 3B like the United States, France, Spain, China, and England took bridging roles in this field, with a centrality of 0.38, 0.25, 0.13, 0.12, and 0.11, respectively (Table 1). However, the collaborative relationship between other countries was relatively weak. Figure 3C shows the annual outputs of the top 10 contributing countries over the past 23 years.

**TABLE 1:** Top five countries/regions that contributed to publications on molecular imaging research in AS.

Sorting by	Rank	Country	Count	Centrality	Year
Frequency					
	1	United States	810	0.38	2000
	2	Peoples R China	283	0.12	2004
	3	Germany	227	0.10	2000
	4	Netherlands	151	0.07	2001
	5	England	130	0.11	2000
Centrality					
	1	United States	810	0.38	2000
	2	France	117	0.25	2000
	3	Spain	50	0.13	2005
	4	Peoples R China	283	0.12	2004
	5	England	130	0.11	2000

### Analysis of co-authorship network of institutions

A total of 443 institutions have been involved in the research on molecular imaging in AS. The top five contributing institutions are summarized in Table 2, of which four were in the United States, Harvard University, Massachusetts General Hospital, Icahn School of Medicine at Mount Sinai, and the University of California System, with a total of 182, 141, 98, and 84 articles, respectively. UDICE-French Research Universities ranked fourth with 85 publications. Furthermore, according to the betweenness centrality ranking, the University of California System was top on the list with 0.20.

**TABLE 2:** Top five institutions that contributed to publications on molecular imaging research in AS.

Sorting by	Rank	Institutions	Country	Frequency		Centrality
Frequency						
	1	Harvard University	United States	182		0.16
	2	Massachusetts General Hospital	United States	141		0.10
	3	Icahn School of Medicine at Mount Sinai	United States of America	98		0.09
	4	UDICE-French Research Universities	France	85		0.10
	5	The University of California System	United States of America	84		0.20
Centrality						
	1	The University of California System	United States	84		0.20
	2	Chinese Academy of Sciences	China	40		0.19
	3	Harvard University	United States	182		0.16
	4	The University of Texas System	United States of America	54		0.16
	5	Massachusetts General Hospital	United States	141		0.10

Year: the earliest year of publication on molecular imaging research in AS of the institution.

**FIGURE 4:**
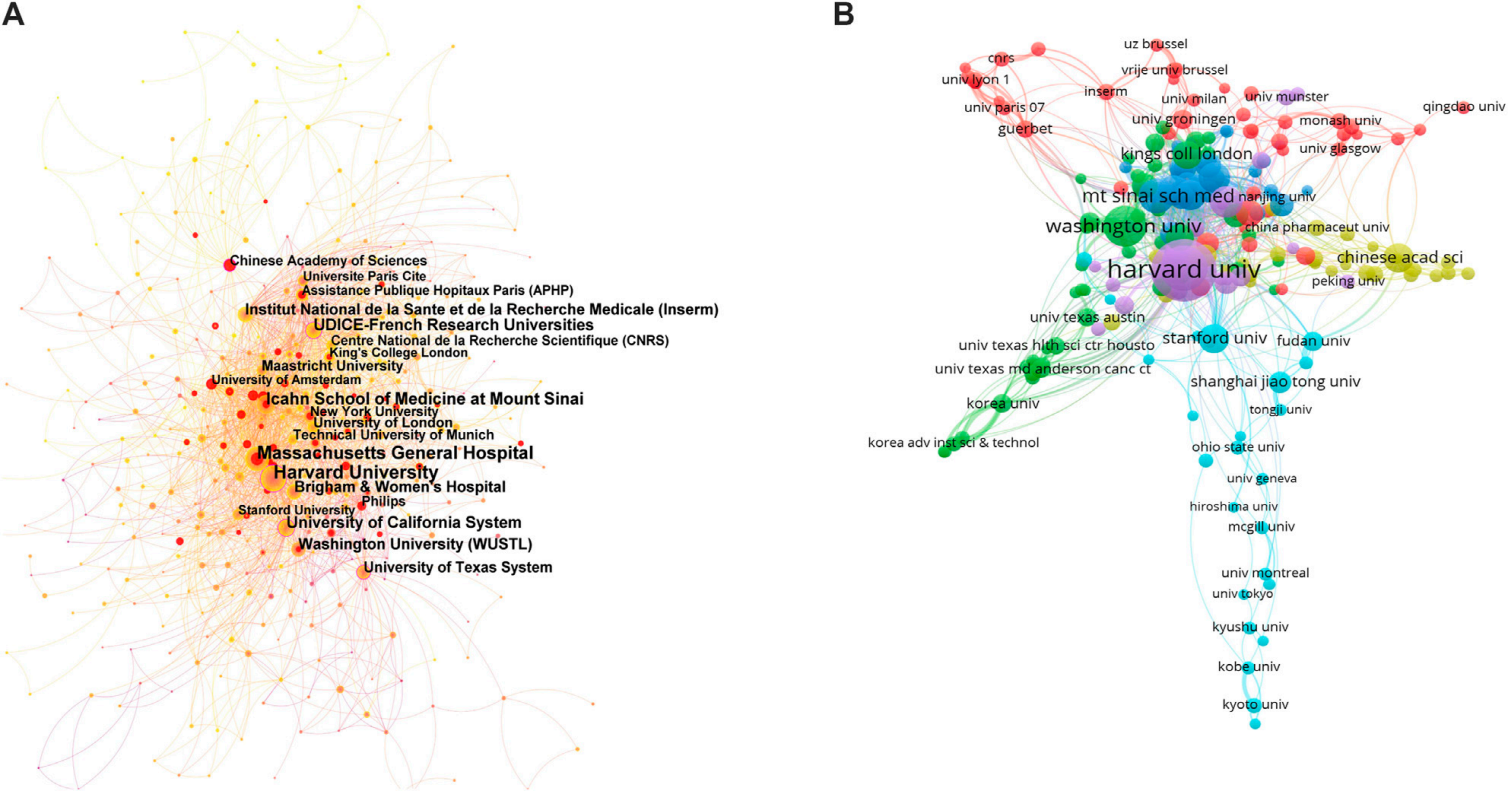
**(A)** The network of collaborating institutions performed with CiteSpace. **(B)** The network of collaborating institutions created with VOSviewer. The size of the node is proportional to the count of an institution’s publications, and links between nodes represent the intensity of collaborations.

Publications from 2000 to 2022 were analyzed with a time slice of 1 year by Citespace, and the top 50 items cited or occurring mostly were selected from each slice. The appearance of two authors’ institutions in the same article is considered one collaboration. Nodes in Figures 4A, B represent institutes, and the co-authorship frequency of institutes corresponds to the size of each node. In Figure 4A, the purple rings indicate the five institutes that were pivotal in this research area. Figure 4B generated by VOSviewer shows the 218 institutions with more than five published papers, among which Harvard University was situated in a central position, and the overall network was relatively loose, which is similar to the Citespace mapping (Density = 0.0229), showing that most institutions out of the United States were scattered and lacked cooperation. 

### Analysis of co-authors and co-cited authors

**FIGURE 5:**
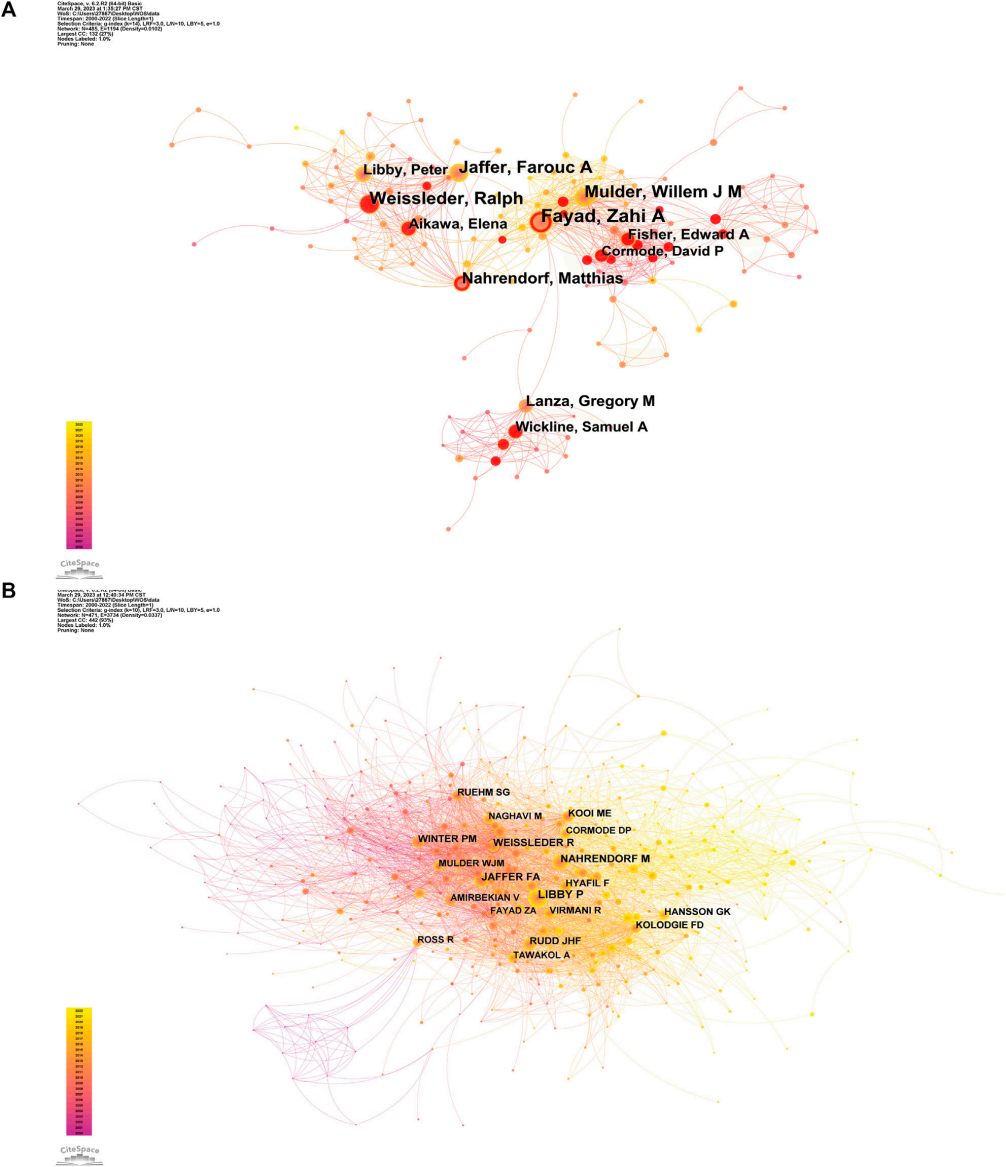
**(A)** The network of co-authors. **(B)** The network of co-cited authors.

A total of 795 authors and 1,020 co-cited authors had participated in the field. The top five most prolific authors and the top five co-cited authors are shown in Table 3. Fayad, ZA ranked first on the co-authors’ list, with 73 articles, followed by Weissleder R, with 51 articles. Whereas all the other authors had a low betweenness centrality (<0.10), which is none of the nodes depicted in the purple ring in Figure 5A. When it came to the co-cited authors’ network map (Figure 5B), Weissleder R was the unique writer with high centrality (0.14) and a citation frequency of 274, showing that his contribution had a significant influence in this domain. Although LIBBY P had the most citations (493), his centrality was oppositely low. The density of the two networks mentioned above was 0.0102 and 0.0337, respectively, which means the scarcity of cooperation between authors.

**TABLE 3:** Top five productive authors and most co-cited authors on molecular imaging research in AS.

Rank	Author	Count	Centrality	Co-cited authors	Count	Centrality
1	Fayad, Zahi A.	73	0.08	Libby P.	493	0.05
2	Weissleder, Ralph	51	0.01	Nahrendorf M.	338	0.08
3	Mulder, Willem J. M.	48	0.02	Jaffer FA	301	0.07
4	Jaffer, Farouc A.	47	0.02	Weissleder R.	274	0.14
5	Nahrendorf, Matthias	34	0.03	Winter PM	233	0.08

### Analysis of the higher-impact journals

Citespace found 785 co-cited journals in this field in the past 23 years. Twelve journals had more than 600 citations. Table 4 shows that Circulation was the most co-cited journal (1,411), followed by Arteriosclerosis Thrombosis and Vascular Biology (1,128), Journal of the American College of Cardiology (JACC) (981), Proceedings of the National Academy of Sciences of the United States (PNAS) (831), and Circulation Research (811). Circulation had the highest IF (39.918), followed by JACC (27.203), while PNAS had the highest H-index (699). Furthermore, all the top five co-cited journals were in the United States and located in Q1 in accordance with the 2021 Journal Citation Report (JCR).

**TABLE 4:** Top five co-cited journals of molecular imaging research in AS in terms of frequency.

Rank	Journal title	Country	Frequency	JCR (2021)	IF (2021)	H-index
1	CIRCULATION	United States	1,411	Q1	39.918	570
2	ARTERIOSCL THROM VAS	United States	1,128	Q1	10.514	251
3	J AM COLL CARDIOL	United States	981	Q1	27.203	394
4	P NATL ACAD SCI United States	United States	831	Q1	12.779	699
5	CIRC RES	United States	811	Q1	23.213	306

**FIGURE 6:**
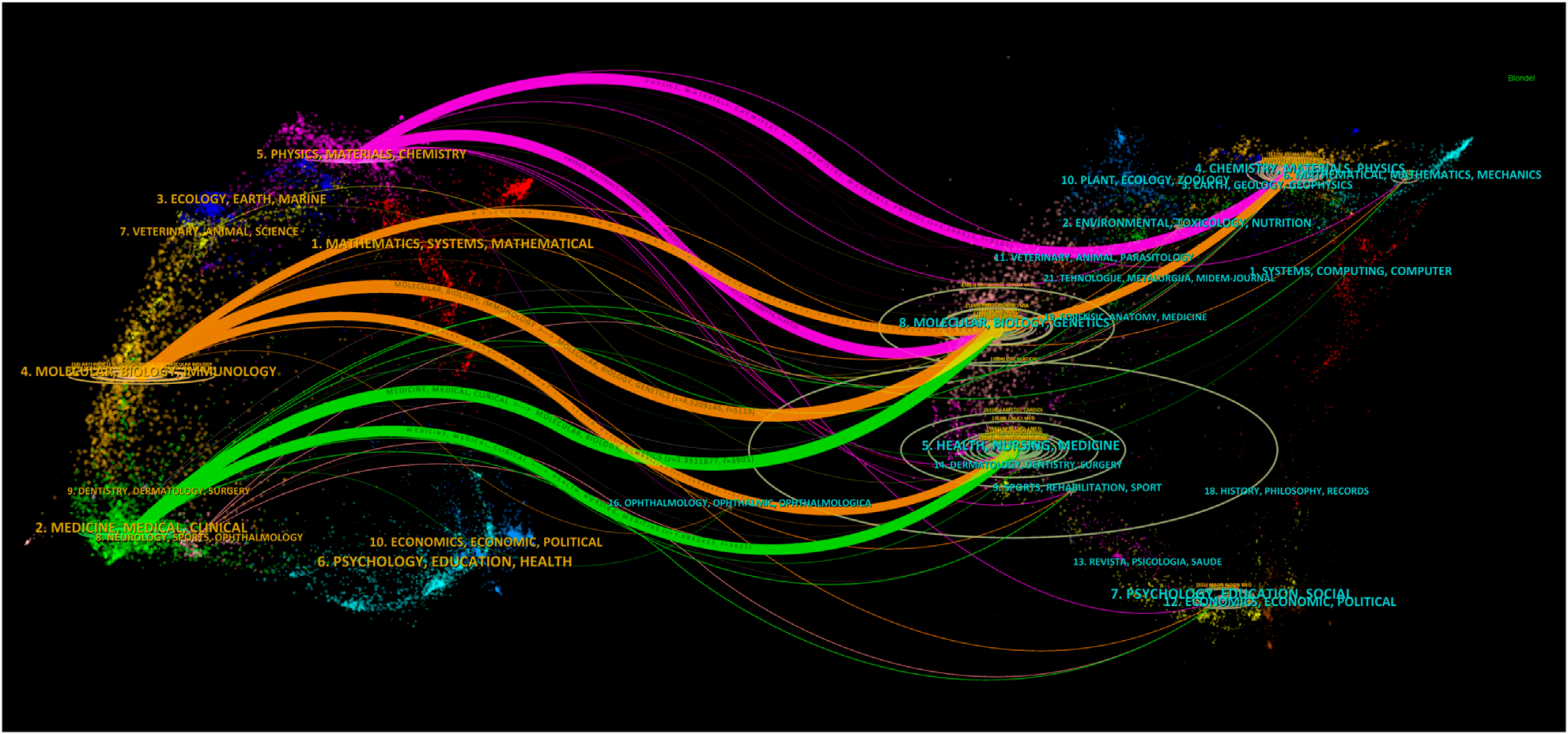
A dual map overlap of journals on molecular imaging research in AS performed with Citespace.

Figure 6 demonstrates the relationship between citing and cited journals by a dual map overlap of journals on molecular imaging research in AS performed with Citespace. It was clear that there were mainly seven citation paths: 1) Physics, Materials, Chemistry—Chemistry, Materials, and Physics; 2) Physics, Materials, Chemistry—Molecular, Biology, and Genetics; 3) Molecular, Biology and Immunology—Chemistry, Materials, and Physics; 4) Molecular, Biology and Immunology—Molecular, Biology, and Genetics; 5) Molecular, Biology and Immunology—Health, Nursing, and Medicine; 6) Medicine, Medical, Clinical—Molecular, Biology, and Genetics; 7) Medicine, Medical, Clinical—Health, Nursing, and Medicine. The citing papers mainly concentrated journals in three fields: 1) Molecular, Biology, and Immunology; 2) Medicine, Medical, and Clinical; 3) Physics, Materials, and Chemistry. Yet the cited papers primarily originated from the journals of 1) Molecular, Biology, and Genetics; 2) Health, Nursing, and Medicine; 3) Chemistry, Materials, and Physics.

### Analysis of keyword co-occurrence and burst

**FIGURE 7:**
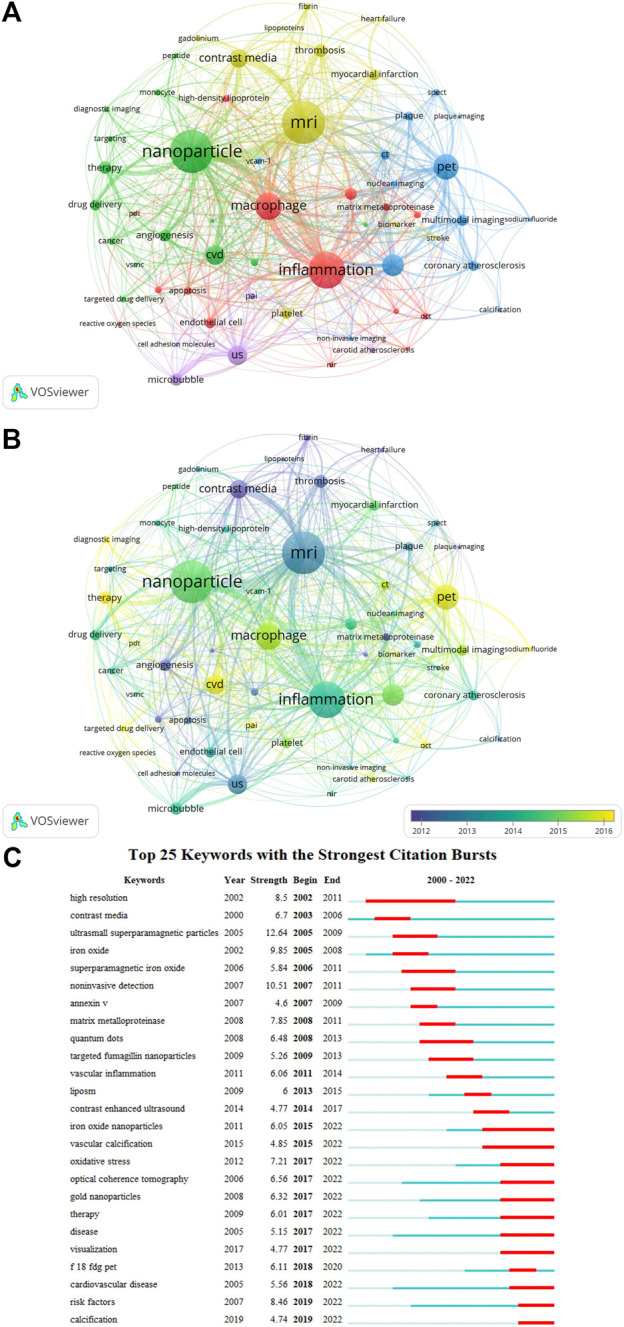
**(A)** The network of keywords co-occurrence conducted by VOSviewer. **(B)** Overlay map of keywords co-occurrence. The nodes in yellow and green represent the keywords appearing chronologically later than those in blue and purple. **(C)** Visualization of the top 25 keywords with the strongest citation bursts from 2000 to 2022 generated by CiteSpace.

There were a total of 2,767 author keywords after merging synonyms and removing irrelevant words in the present research, and 64 keywords with a frequency of at least 10 times are shown in Figures 7A, B. Among these, the top 10 keywords of molecular imaging research in AS according to occurring frequency are listed in Table 5. Nanoparticle, magnetic resonance imaging (MRI), and inflammation were the top three, with 277, 275, and 218 appearances, respectively, and some of the remainder were concerning imaging approaches and materials such as positron emission tomography (PET), ultrasonography (US), and contrast media. Others were related to relevant diseases and their pathophysiologies such as cardiovascular disease (CVD), vulnerable plaque, macrophage, and thrombosis.

**TABLE 5:** Top 10 keywords of molecular imaging research in AS in terms of frequency.

Rank	Keyword	Frequency	TSL
1	Nanoparticle	277	429
2	Magnetic resonance imaging	275	469
3	Inflammation	218	354
4	Macrophage	143	239
5	Positron emission tomography	130	203
6	Vulnerable plaque	106	148
7	Cardiovascular disease	92	135
8	Ultrasonography	90	143
9	Contrast media	84	157
10	Thrombosis	51	97

TLS: total link strength.

Figure 7A demonstrates that all the research could be clustered into five clusters via co-occurrence analysis of keywords: 1) Cluster in green (nanoparticle-related studies), 2) Cluster in yellowish green (MRI-related studies), 3) Cluster in red (Inflammation -related studies), 4) Cluster in blue (PET-related studies), and 5) Cluster in purple (US-related studies). The second largest nodes in each cluster that represents the primary keywords were cardiovascular disease, contrast media, macrophage, vulnerable plaque, and microbubble, respectively, suggesting the hotspots each cluster focused on so far. The overlay map of keywords’ co-occurrence indicates the evolution of keywords over time. The nodes in yellow and green represent the keywords appearing chronologically later than those in blue and purple. As shown in Figure 7B, sodium fluoride ⁃PET, photoacoustic imaging (PAI), reactive oxygen species (ROS), optical coherence tomography (OCT), CVD, targeted drug delivery, and therapy colored with yellow were identified as the emerging trends in the coming years.

Moreover, we carried out keyword burst detection based on Citespace by determining keywords increasing suddenly in frequency in a short time, which can also reflect the study hotspots over time and forecast the emerging trends. It can be seen in Figure 7C that the research trends in the past 23 years had evolved from “high resolution” (2002) and “contrast media” (2003) to “18F-FDG PET” (2020), “CVD” (2022), “OCT” (2022), and “therapy” (2022), similar to the results of the overlay map of keywords co-occurrence analysis. In addition, the keyword with the strongest strength of citation bursts was “ultrasmall superparamagnetic particle” (12.64) in 2005. The keyword with the longest bursting duration was “high resolution”, up to 10 years, which implied that these topics had received the longest attention.

### Analysis of co-citation reference and reference burst

Table 6 shows a list of the top five most co-cited papers, all of which were published from 2005 to 2008 and had more than 50 citations, with two published in Circulation. Especially, the top one with a citation frequency of 81 was titled “Non-invasive vascular cell adhesion molecule-1 imaging identifies inflammatory activation of cells in atherosclerosis” published in 2006 (Nahrendorf et al., 2006). This article demonstrated that a novel VCAM-1-targeted agent that combined with MRI could non-invasively detect inflammation in subclinical atherosclerosis.

**TABLE 6:** Top five co-citation papers of molecular imaging research in AS in terms of frequency.

Rank	Cited reference	Frequency	Centrality	Year
1	Nahrendorf M, 2006, CIRCULATION, V114, P1504, DOI 10.1161/CIRCULATIONAHA.106.646380	81	0.14	2006
2	Amirbekian V, 2007, P NATL ACAD SCI United States, V104, P961, DOI 10.1073/pnas.0606281104	80	0.11	2007
3	Sanz J, 2008, NATURE, V451, P953, DOI 10.1038/nature06803	70	0.01	2008
4	Nahrendorf M, 2008, CIRCULATION, V117, P379, DOI 10.1161/CIRCULATIONAHA.107.741181	63	0.10	2008
5	Kelly KA, 2005, CIRC RES, V96, P327, DOI 10.1161/01.RES.0000155722.17881.dd	59	0.04	2005

**FIGURE 8:**
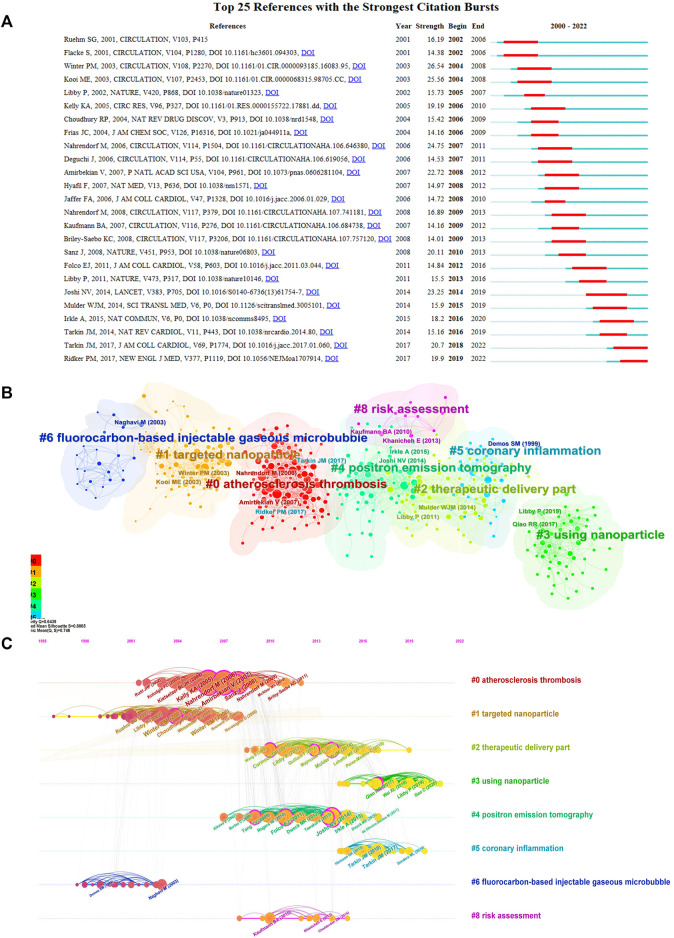
**(A)** CiteSpace visualization of the top 25 references with the strongest citation bursts from 2000 to 2022. **(B)** Citespace visualization of cluster view of co-citation references labeled by subject categories. **(C)** Citespace visualization of timeline view of co-citation references. The nodes represent co-citation references, and the lines with different colors between the nodes indicate time evolution.

References with citation bursts indicate research interest with abrupt increments in the field. The strength of the citation burst is used to estimate the innovation of the study outcomes. The top 25 papers with the strongest cation bursts are illustrated in Figure 8A. The explosion of citations in this domain began in 2002, and the highest-ranked research with a burst strength of 26.54 was published in Circulation by Winter PM (Winter et al., 2003), which used alpha(v)beta3-Integrin-targeted, paramagnetic nanoparticles to detect the neovasculature of abdominal aorta plaques and proved that molecular imaging might offer a method for defining the burden and evolution of atherosclerosis as well as the response to therapies. From then on, non-invasive detection, iron oxide nanoparticles, gold nanoparticles, therapy, cardiovascular disease, risk factors, and sodium fluoride ⁃PET had attracted more attention from academia, which might be the potential Frontier in the future, resembling the outcomes of keywords burst detection.

As shown in Figure 8B, the co-citation references analysis identified eight clusters with a good homogeneity network (Q = 0.6439) and highly convincing clustering (S = 0.8865), which were labeled with title words. The largest cluster (#0) had 225 members and a silhouette value of 0.887, which was labeled as _atherosclerosis thrombosis_ by LLR and atherosclerotic plaque by LSI. The second largest cluster had 186 members and a silhouette value of 0.928, and it was labeled as _targeted nanoparticle by LLR and _atherosclerotic plaque by LSI. The major citing article titled “Molecular Imaging by Cardiovascular MR” was written by Cyrus et al. (2007). Figure 8C illustrates the eight clusters in a timeline visualization, and each one could reflect the evolution of the molecular imaging sub-domain in AS over time. The latest hot themes in this field were “therapeutic delivery part”, “using nanoparticles”, “PET”, and “coronary inflammation”.

## Discussion

With the great progress of nanotechnology and bioengineering, massive numbers of research continue to explore the world of molecular imaging in the precise diagnosis and treatment of various diseases over the past 23 years, encompassing atherosclerosis. Hence, this current study employed two widely used bibliometric analysis applications (CiteSpace and VOSviewer) to analyze the co-authorship of nations, authors, and organizations, co-citation of authors, papers, and journals in detail and grasped a comprehensive understanding as well as the hotspots and frontiers in this field.

### Overview of molecular imaging in AS

In 2000, there were only eight papers published in this field since the concept of Molecular Imaging was first proposed in 1999. However, in 2010, the number of publications surged to 108. From 2011 to 2022, molecular imaging research in AS has undergone a stable development period accompanied by a wave-like uplift trend. Moreover, all scientific literature had been highly cited, which indicates that more investigators are focused on the domain, and the number of papers is likely to increase in the coming years.

The United States was the leading country and institution in terms of the number and centrality of publications in this domain, and Harvard University published the largest number of articles. The University of California System was top on the list of betweenness centrality with 0.20. Moreover, the number of publications from the United States occupied nearly half of the total papers, 810 papers, far more than other countries. Remarkably, though China engaged late in this field from 2004, papers issued by China had a burst in the last 3 years, indicating that more and more researchers in China participate in molecular imaging research in AS. Moreover, the centrality of China had reached 0.12, meaning that China began to collaborate closely with other authoritative countries in this field, especially the United States, and had published extensive and lucubrate research, possessing a certain influence in this field. Among the top five most high-yielding institutions, four were in the United States, and Harvard University had made the most outstanding contribution in this field.

The results of the co-authorship analysis showed Fayad, ZA from New York University as the most productive author and he was also top on the centrality list, and it is known to all that he is a physician with a high H-index (125) and devoted to the research on imaging atherosclerosis. Unfortunately, the centrality of all the authors was less than 0.1, suggesting that even though lots of scholars were involved in this area, they were relatively dispersal. It is worth noting that Fayad, ZA and his team’s current research is in the development and use of CMR (Calcagno et al., 2023) and fast computed tomography (CT) to characterize the elements leading to atherosclerotic plaque rupture (Achenbach et al., 2022; Senders et al., 2022) and the definition of the components of plaque most active in initiating thrombosis (Senders et al., 2018). Meanwhile, as for the co-cited authors’ analysis, LIBBY P from Harvard Medical School and Brigham and Women’s Hospital ranked first with a citation frequency of 493, and he is a cardiologist committed to clinical and basal research focus on the role of inflammation in vascular diseases such as atherosclerosis, and he has published large numbers of papers from 1995 up to the present (Libby and Everett, 2019; Ridker et al., 2021; Stone et al., 2022). Interestingly, Weissleder R from Harvard University was a unique writer with high centrality (0.14), and he was the person who first proposed the concept of “Molecular Imaging” and was named by Thomson Reuters as one of the “The World’s Most Influential Scientific Minds”. Furthermore, his research group developed novel molecular technologies for non-invasive imaging of cellular function so as to create next-generation diagnostics and therapeutics in AS (Cremer et al., 2020; Nahrendorf et al., 2020) and cancers (Dosta et al., 2023). Significantly, all the most productive and co-cited authors mentioned above were from the United States. Whereas the network with low density showed that most scholars in the field were scattered and scholars from emerging countries should carry on more communication and cooperation with American scholars.

As is well-known, citation frequency, JCR category, IF, and H-index are effective indicators for evaluating the quality of journals. It was found that all the top five co-cited journals with high IF (each greater than 10) and H-index (each no less than 250) were in the United States and located in Q1, which had formed a reliable theoretical basis for this research field. Among them, Circulation had the most citations, indicating its important role in this field. It is foreseeable that more researchers interested in this domain would prefer to submit their articles in the above influential journals in the future.

### Knowledge structure, hotspots, and emerging trends of molecular imaging in AS

Co-occurrence analysis of keywords clarifies the research hot topics in the study, and clustering can display the knowledge structure. Furthermore, overlay view and citations bursts detection are two of the central methods for tracing the evolution of science (Yuan et al., 2021). The treatment of cardio-cerebrovascular diseases caused by AS is a major global health concern (De Luca et al., 2022), and the application of molecular imaging will have a profound impact on the early diagnosis and precise treatment of AS (Lin et al., 2021; Wang et al., 2021). Figure 7A shows that the research was divided into five clusters which constructed the knowledge structures in this field:

(1) Nanoparticle-related studies. As the largest cluster, nanoparticles which are of top importance in molecular imaging as contrast agents or/and nanomedicine have been widely developed for multimodal molecular imaging and atherosclerosis therapy (Padmanabhan et al., 2016; Atukorale et al., 2017; Evans et al., 2020), NaF (Singh et al., 2023), and small peptide targeting ligands (Moccetti et al., 2018) and other tracers are emerging.
(2) MRI-related studies. MRI combined with nanoprobes as a non-invasive imaging technique with high spatial resolution and excellent soft tissue contrast can determine component information of plaque and identify vulnerable plaques (Gitsioudis et al., 2017; Rashid et al., 2018; Hajhosseiny et al., 2019; Park et al., 2022).
(3) Inflammation-related studies. Since atherosclerosis is a chronic inflammatory disease, vulnerable plaques are also associated with inflammation; many studies focus on targeting vascular inflammatory cells to study the effects and mechanisms of inflammation inhibition or reversal (Liu and Woodard, 2019; Xie et al., 2020; Sammartino et al., 2023).
(4) PET-related studies. PET is widely used in the study of cardio-cerebrovascular diseases because of its superior sensitivity, functional detection, and non-invasive characteristics and provides a new molecular imaging approach to identify high-risk patients (Rosenbaum et al., 2012; Kitagawa et al., 2017; Piri et al., 2020).
(5) US-related studies. Ultrasound is the most commonly used clinical imaging technique that can rapidly measure the size of arterial plaques, IMT, and even the ulceration of plaque without radiation. Ultrasound with nanobubble or nanodroplet contrast agents can greatly improve the potential for early diagnosis and targeted therapy of AS (Moccetti et al., 2018; Liu et al., 2022; Punjabi et al., 2019; Rykaczewska et al., 2022).

Figure 7A demonstrates that “cardiovascular disease” which is often caused by coronary atherosclerosis and its detection by PET/CT (Paydary et al., 2021), PET/MRI (Wurster et al., 2022), and OCT (Liu et al., 2021; Munoz-Ortiz et al., 2022) has been widely studied. “Macrophage,” “vulnerable plaque” which refers to plaque that is unstable and thrombotic-prone, including ruptured plaque, erosive plaque, and partially calcified nodular lesion (Strauss and Narula, 2017), “microbubble,” and “contrast media” were identified as the hotspot of each cluster. Moreover, as shown in Figure 7B, “NaF-PET” as a kind of novel PET (Singh et al., 2023), “ROS” as a key factor in the formation of atherosclerosis (Xu et al., 2022), “photoacoustic imaging (PAI)” as a kind of dual-modality imaging method (Ma et al., 2022), and “targeted drug delivery” as a newly developed approach of therapy (Boersma et al., 2022) were identified as the emerging trends in the coming years.

Analysis of co-citation references is a significant method to measure the most influential articles in a certain research area n(Gao et al., 2020). As shown in Table 6, all of the top five articles were published prior to 2010, and the most highly co-cited article was written by Nahrendorf M (Nahrendorf et al., 2006), who developed a novel, second-generation VCAM-1-targeted agent to enable real-time detection of VCAM-1 expression in experimental atherosclerosis *in vivo*, so as to non-invasively detect inflammation in early, subclinical atherosclerosis. Obviously, with the development of materials science and biomedical engineering, the exploitation and application of large amounts of nanoparticles have become an important research field. Based on nanoparticles, a lot of prospective and extensive research was conducted. In Figure 8B, studies with similar title words are inclined to be concentrated in a cluster, indicating that articles about similar topics are usually cited together.

Through visualization of the timeline of the co-citation references clusters (Figure 8C), which reflects the dynamic changes of the hotspots and research trends of each cluster in different stages, it could be seen that the earliest research concentrated on “high-resolution” (Yuan et al., 2002; Blake et al., 2003), and the latest research hotspots were “therapeutic delivery part” (Li et al., 2022b), “coronary inflammation” (Song et al., 2021), and “using nanoparticle” (Tu et al., 2022), indicating that the research focus has changed from diagnosis to therapy, and the research topic turned from anatomical structure imaging to functional molecular imaging of pathological processes.

### Challenges of molecular imaging in AS

It can be seen from the above that the research of molecular imaging in AS has obtained masses of achievements, early diagnosis, risk predictions, and targeted precise treatment will be the directions in the future. However, to date, most of the studies are still in the experimental stage as the lack of standardization of locally-enriched concentration, physical distribution, drug release, biosafety assessment, and their conversion to the clinic needs to be verified by further studies.

## Limitations

Although this current research was performed by two bibliometric applications juxtaposed with one online scientometric platform, which provided more thorough and objective outcomes, there are still some limitations. First of all, only the articles and reviews in English from the SCIE database were included; certain significant research in other databases such as Google Scholar and Scopus or in other languages might be neglected. Hence, the integrity of the data is insufficient, and the report findings may be influenced. Nevertheless, SCIE, as the world’s most powerful database in the medical field is the most commonly used database in scientometric analysis, and a great majority of scientific articles are published in English. Therefore, the results of this study still have some referential significance. Secondly, in general, there is a time delay in citations of high-quality papers published recently. In addition, some keywords and institutions’ names have different expressions, which may have an impact on the clustering analysis even after our manual inspection procedures. Last but not least, similar to other bibliometric reports (Brandt et al., 2019), this study may ignore the abundant semantic information contained in the sample literature, and the perspective of analyzing the evolution of this field is relatively single.

## Conclusion

Taken together, molecular imaging research in atherosclerosis has attracted extensive attention in academia. The United States has always been the research center in this field and the top five high-yield developed countries collaborated closely in the past 23 years, and the academic impact of China has been emerging in recent years; however, the cooperation intensity of developing countries and communication among authors still need to be enhanced. High-resolution MR and applications of molecular imaging in cardio-cerebrovascular diseases have been core research topics throughout the field. Particularly, 18F-NaF⁃PET, nanoparticle (including targeted therapeutic delivery part), ROS and oxidative stress, multimodal imaging, and inflammation of atherosclerosis may be the potential trends in the coming years though there are still many challenges in molecular target selection, molecular probe development, and clinical transformation in this field. Moreover, these findings of the present research may help funding agencies and researchers determine future directions.

## Data Availability

The original contributions presented in the study are included in the article/supplementary material, further inquiries can be directed to the corresponding author.

## References

[B1] Achenbach S. Fuchs F. Goncalves A. Kaiser-Albers C. Ali Z. A. Bengel F. M. (2022). Non-invasive imaging as the cornerstone of cardiovascular precision medicine. Eur. Heart J. Cardiovasc Imaging 23, 465–475. 10.1093/ehjci/jeab287 35048106 PMC13376137

[B2] Ahmed M. Tegnebratt T. Tran T. A. Lu L. Damberg P. Gistera A. (2020). Molecular imaging of inflammation in a mouse model of atherosclerosis using a zirconium-89-labeled probe. Int. J. Nanomedicine 15, 6137–6152. 10.2147/ijn.s256395 32884268 PMC7434576

[B3] Al Rifai M. Blaha M. J. Nambi V. Shea S. J. C. Michos E. D. Blumenthal R. S. (2022). Determinants of incident atherosclerotic cardiovascular disease events among those with absent coronary artery calcium: Multi-ethnic study of atherosclerosis. Circulation 145, 259–267. 10.1161/circulationaha.121.056705 34879218 PMC8792296

[B4] Atukorale P. U. Covarrubias G. Bauer L. Karathanasis E. (2017). Vascular targeting of nanoparticles for molecular imaging of diseased endothelium. Adv. Drug Deliv. Rev. 113, 141–156. 10.1016/j.addr.2016.09.006 27639317 PMC5352558

[B5] Bala G. Cosyns B. (2014). Recent advances in visualizing vulnerable plaque: Focus on noninvasive molecular imaging. Curr. Cardiol. Rep. 16, 520. 10.1007/s11886-014-0520-5 25059464

[B6] Blake G. J. Ostfeld R. J. Yucel E. K. Varo N. Schonbeck U. Blake M. A. (2003). Soluble CD40 ligand levels indicate lipid accumulation in carotid atheroma: An *in vivo* study with high-resolution MRI. Arterioscler. Thromb. Vasc. Biol. 23, e11–e14. 10.1161/01.atv.0000050143.22910.62 12524242

[B7] Boersma B. Moller K. Wehl L. Puddinu V. Huard A. Fauteux-Daniel S. (2022). Inhibition of IL-1 beta release from macrophages targeted with necrosulfonamide-loaded porous nanoparticles. J. Control. Release 351, 989–1002. 10.1016/j.jconrel.2022.09.063 36202154

[B8] Borne Y. Fagerberg B. Persson M. Ostling G. Soderholm M. Hedblad B. (2017). Cadmium, carotid atherosclerosis, and incidence of ischemic stroke. J. Am. Heart Assoc. 6, e006415. 10.1161/jaha.117.006415 29197829 PMC5778998

[B9] Brandt J. S. Hadaya O. Schuster M. Rosen T. Sauer M. V. Ananth C. V. (2019). A bibliometric analysis of top-cited journal articles in obstetrics and gynecology. JAMA Netw. Open 2, e1918007. 10.1001/jamanetworkopen.2019.18007 31860106 PMC6991228

[B10] Calcagno C. David J. A. Motaal A. G. Coolen B. F. Beldman T. Corbin A. (2023). Self-gated, dynamic contrast-enhanced magnetic resonance imaging with compressed-sensing reconstruction for evaluating endothelial permeability in the aortic root of atherosclerotic mice. NMR Biomed. 36, e4823. 10.1002/nbm.4823 36031706 PMC10078106

[B11] Chen C. Lou Y. Li X. Y. Lv Z. T. Zhang L. Q. Mao W. (2020). Mapping current research and identifying hotspots on mesenchymal stem cells in cardiovascular disease. Stem Cell. Res. Ther. 11, 498. 10.1186/s13287-020-02009-7 33239082 PMC7687818

[B12] Cheng K. Guo Q. Shen Z. Yang W. Wang Y. Sun Z. (2022). Bibliometric analysis of global research on cancer photodynamic therapy: Focus on nano-related research. Front. Pharmacol. 13, 927219. 10.3389/fphar.2022.927219 35784740 PMC9243586

[B13] Colantonio L. D. Booth J. N., 3R. D. Bress A. P. Whelton P. K. Shimbo D. Levitan E. B. (2018). 2017 ACC/AHA blood pressure treatment guideline recommendations and cardiovascular risk. J. Am. Coll. Cardiol. 72, 1187–1197. 10.1016/j.jacc.2018.05.074 30189994 PMC6346270

[B14] Cremer S. Schloss M. J. Vinegoni C. Foy B. H. Zhang S. Rohde D. (2020). Diminished reactive hematopoiesis and cardiac inflammation in a mouse model of recurrent myocardial infarction. J. Am. Coll. Cardiol. 75, 901–915. 10.1016/j.jacc.2019.12.056 32130926 PMC7254576

[B15] Cyrus T. Lanza G. M. Wickline S. A. (2007). Molecular imaging by cardiovascular MR. J. Cardiovasc Magn. Reson 9, 827–843. 10.1080/10976640701693766 18066742

[B16] de Luca L. Temporelli P. L. Colivicchi F. Gonzini L. Fasano M. L. Pantaleoni M. (2022). Clinical impact and prognostic role of triglyceride to high-density lipoprotein cholesterol ratio in patients with chronic coronary syndromes at very high risk: Insights from the START study. Front. Cardiovasc Med. 9, 874087. 10.3389/fcvm.2022.874087 35498014 PMC9043517

[B17] Dosta P. Puigmal N. Cryer A. M. Rodriguez A. L. Scott E. Weissleder R. (2023). Polymeric microneedles enable simultaneous delivery of cancer immunomodulatory drugs and detection of skin biomarkers. Theranostics 13, 1–15. 10.7150/thno.73966 36593949 PMC9800729

[B18] Douma K. Prinzen L. Slaaf D. W. Reutelingsperger C. P. Biessen E. A. Hackeng T. M. (2009). Nanoparticles for optical molecular imaging of atherosclerosis. Small 5, 544–557. 10.1002/smll.200801079 19226595

[B19] Duan H. Iagaru A. Aparici C. M. (2022). Radiotheranostics - precision medicine in nuclear medicine and molecular imaging. Nanotheranostics 6, 103–117. 10.7150/ntno.64141 34976584 PMC8671964

[B20] Dweck M. R. Doris M. K. Motwani M. Adamson P. D. Slomka P. Dey D. (2016). Imaging of coronary atherosclerosis - evolution towards new treatment strategies. Nat. Rev. Cardiol. 13, 533–548. 10.1038/nrcardio.2016.79 27226154

[B21] Evans R. J. Lavin B. Phinikaridou A. Chooi K. Y. Mohri Z. Wong E. (2020). Targeted molecular iron oxide contrast agents for imaging atherosclerotic plaque. Nanotheranostics 4, 184–194. 10.7150/ntno.44712 32637296 PMC7332796

[B22] Fan J. Gao Y. Zhao N. Dai R. Zhang H. Feng X. (2020). Bibliometric analysis on COVID-19: A comparison of research between English and Chinese studies. Front. Public Health 8, 477. 10.3389/fpubh.2020.00477 32923422 PMC7456831

[B23] Gao Q. Zhang C. Wang J. Wei Q. Wei Q. Miyamoto A. (2020). The top 100 highly cited articles on osteoporosis from 1990 to 2019: A bibliometric and visualized analysis. Arch. Osteoporos. 15, 144. 10.1007/s11657-020-0705-z 32935223

[B24] Gitsioudis G. Chatzizisis Y. S. Wolf P. Missiou A. Antoniadis A. P. Mitsouras D. (2017). Combined non-invasive assessment of endothelial shear stress and molecular imaging of inflammation for the prediction of inflamed plaque in hyperlipidaemic rabbit aortas. Eur. Heart J. Cardiovasc Imaging 18, 19–30. 10.1093/ehjci/jew048 27013245 PMC5217740

[B26] Hajhosseiny R. Bahaei T. S. Prieto C. Botnar R. M. (2019). Molecular and nonmolecular magnetic resonance coronary and carotid imaging. Arterioscler. Thromb. Vasc. Biol. 39, 569–582. 10.1161/atvbaha.118.311754 30760017

[B28] Hughes D. J. Subesinghe M. Taylor B. Bille A. Spicer J. Papa S. (2022). 18 F FDG PET/CT and novel molecular imaging for directing immunotherapy in cancer. Radiology 304, 246–264. 10.1148/radiol.212481 35762888

[B29] Ioannidis J. P. A. Baas J. Klavans R. Boyack K. W. (2019). A standardized citation metrics author database annotated for scientific field. PLoS Biol. 17, e3000384. 10.1371/journal.pbio.3000384 31404057 PMC6699798

[B31] Kitagawa T. Yamamoto H. Toshimitsu S. Sasaki K. Senoo A. Kubo Y. (2017). 18 F-sodium fluoride positron emission tomography for molecular imaging of coronary atherosclerosis based on computed tomography analysis. Atherosclerosis 263, 385–392. 10.1016/j.atherosclerosis.2017.04.024 28528743

[B32] Lackner A. Fathalla S. Nayyeri M. Behrend A. Manthey R. Auer S. (2021). Analysing the evolution of computer science events leveraging a scholarly knowledge graph: A scientometrics study of top-ranked events in the past decade. Scientometrics 126, 8129–8151. 10.1007/s11192-021-04072-0 34276109 PMC8272613

[B33] Li B. Hu K. Lysenko V. Khan K. Y. Wang Y. Jiang Y. (2022a). A scientometric analysis of agricultural pollution by using bibliometric software VoSViewer and Histcite. Environ. Sci. Pollut. Res. Int. 29, 37882–37893. 10.1007/s11356-022-18491-w 35067891

[B34] Li X. X. Qi H. Z. Cui W. G. Wang Z. B. Fu X. X. Li T. X. (2022b). Recent advances in targeted delivery of non-coding RNA-based therapeutics for atherosclerosis. Mol. Ther. 30, 3118–3132. 10.1016/j.ymthe.2022.07.018 35918894 PMC9552813

[B35] Libby P. Everett B. M. (2019). Novel antiatherosclerotic therapies. Arterioscler. Thromb. Vasc. Biol. 39, 538–545. 10.1161/atvbaha.118.310958 30816799 PMC6436984

[B36] Lin L. Xie Z. Xu M. Wang Y. Li S. Yang N. (2021). IVUS\IVPA hybrid intravascular molecular imaging of angiogenesis in atherosclerotic plaques via RGDfk peptide-targeted nanoprobes. Photoacoustics 22, 100262. 10.1016/j.pacs.2021.100262 33868920 PMC8040266

[B37] Liu N. Chen X. Kimm M. A. Stechele M. Chen X. L. Zhang Z. M. (2021). *In vivo* optical molecular imaging of inflammation and immunity. J. Mol. Medicine-Jmm 99, 1385–1398. 10.1007/s00109-021-02115-w 34272967

[B38] Liu Y. Woodard P. K. (2019). Chemokine receptors: Key for molecular imaging of inflammation in atherosclerosis. J. Nucl. Cardiol. 26, 1179–1181. 10.1007/s12350-018-1248-1 29516368 PMC6128785

[B94] Liu F. Mao Y. Yan J. Sun Y. Xie Z. Li F. (2022). Bionic microbubble neutrophil composite for inflammation-responsive atherosclerotic vulnerable plaque pluripotent intervention. Research (Wash D C) 2022, 9830627. 35711673 10.34133/2022/9830627 PMC9188677

[B39] Lv R. Wang L. Maehara A. Guo X. Zheng J. Samady H. (2022). Image-based biomechanical modeling for coronary atherosclerotic plaque progression and vulnerability prediction. Int. J. Cardiol. 352, 1–8. 10.1016/j.ijcard.2022.02.005 35149139

[B40] Ma B. Xiao Y. Lv Q. Li G. Wang Y. Fu G. (2022). Targeting theranostics of atherosclerosis by dual-responsive nanoplatform via photoacoustic imaging and three-in-one integrated lipid management. Adv. Mater 35, e2206129. 10.1002/adma.202206129 36394179

[B42] Mensah G. A. Roth G. A. Fuster V. (2019). The global burden of cardiovascular diseases and risk factors: 2020 and beyond. J. Am. Coll. Cardiol. 74, 2529–2532. 10.1016/j.jacc.2019.10.009 31727292

[B43] Miao X. Sha T. Zhang W. Zhou H. Qiu C. Deng H. (2022). Liver fibrosis assessment by viewing sinusoidal capillarization: US molecular imaging versus two-dimensional shear-wave elastography in rats. Radiology 304, 473–482. 10.1148/radiol.212325 35503015

[B44] Moccetti F. Weinkauf C. C. Davidson B. P. Belcik J. T. Marinelli E. R. Unger E. (2018). Ultrasound molecular imaging of atherosclerosis using small-peptide targeting ligands against endothelial markers of inflammation and oxidative stress. Ultrasound Med. Biol. 44, 1155–1163. 10.1016/j.ultrasmedbio.2018.01.001 29548756 PMC12871067

[B45] Mohanta S. K. Peng L. Li Y. Lu S. Sun T. Carnevale L. (2022). Neuroimmune cardiovascular interfaces control atherosclerosis. Nature 605, 152–159. 10.1038/s41586-022-04673-6 35477759

[B46] Munoz-Ortiz T. Hu J. Sanz-Rodriguez F. Ortgies D. H. Jaque D. Mendez-Gonzalez D. (2022). Optical detection of atherosclerosis at molecular level by optical coherence tomography: An *in vitro* study. Nanomedicine-Nanotechnology Biol. Med. 43, 102556. 10.1016/j.nano.2022.102556 35390527

[B47] Nahrendorf M. Hoyer F. F. Meerwaldt A. E. van Leent M. M. T. Senders M. L. Calcagno C. (2020). Imaging cardiovascular and lung macrophages with the positron emission tomography sensor (64)Cu-macrin in mice, rabbits, and pigs. Circ. Cardiovasc Imaging 13, e010586. 10.1161/circimaging.120.010586 33076700 PMC7583675

[B48] Nahrendorf M. Jaffer F. A. Kelly K. A. Sosnovik D. E. Aikawa E. Libby P. (2006). Noninvasive vascular cell adhesion molecule-1 imaging identifies inflammatory activation of cells in atherosclerosis. Circulation 114, 1504–1511. 10.1161/circulationaha.106.646380 17000904

[B50] Novo M. S. Geracitano L. A. Henning P. (2013). Padrão de relacionamento entre nanociências, saúde e biologia: Um levantamento histórico utilizando o programa Citespace. Hist. Cienc. Saude Manguinhos 20, 1657–1670. 10.1590/s0104-59702013005000008 23827968

[B52] Padmanabhan P. Kumar A. Kumar S. Chaudhary R. K. Gulyas B. (2016). Nanoparticles in practice for molecular-imaging applications: An overview. Acta Biomater. 41, 1–16. 10.1016/j.actbio.2016.06.003 27265153

[B53] Park S. J. Chan W. Y. Ng M. Chung Y. C. Chong T. T. Bhakoo K. (2022). Development of molecular magnetic resonance imaging tools for longitudinal tracking of carotid atherosclerotic disease using fast imaging with steady-state precession. Transl. Stroke Res. 10.1007/s12975-022-01067-8 PMC10159972 35856131

[B54] Paydary K. Revheim M. E. Emamzadehfard S. Gholami S. Pourhassan S. Werner T. J. (2021). Quantitative thoracic aorta calcification assessment by (18)F-NaF PET/CT and its correlation with atherosclerotic cardiovascular disorders and increasing age. Eur. Radiol. 31, 785–794. 10.1007/s00330-020-07133-9 32870396

[B55] Piri R. Gerke O. Hoilund-Carlsen P. F. (2020). Molecular imaging of carotid artery atherosclerosis with PET: A systematic review. Eur. J. Nucl. Med. Mol. Imaging 47, 2016–2025. 10.1007/s00259-019-04622-y 31786626

[B95] Punjabi M. Xu L. Ochoa-Espinosa A. Kosareva A. wolff T. Murtaja A. (2019). Ultrasound molecular imaging of atherosclerosis with nanobodies: Translatable microbubble targeting murine and human VCAM (vascular cell adhesion molecule) 1. Arterioscler. Thromb. Vasc. Biol. 39, 2520–2530. 31597443 10.1161/ATVBAHA.119.313088

[B57] Rashid I. Maghzal G. J. Chen Y. C. Cheng D. Talib J. Newington D. (2018). Myeloperoxidase is a potential molecular imaging and therapeutic target for the identification and stabilization of high-risk atherosclerotic plaque. Eur. Heart J. 39, 3301–3310. 10.1093/eurheartj/ehy419 30219874

[B58] Ridker P. M. Devalaraja M. Baeres F. M. M. Engelmann M. D. M. Hovingh G. K. Ivkovic M. (2021). IL-6 inhibition with ziltivekimab in patients at high atherosclerotic risk (RESCUE): A double-blind, randomised, placebo-controlled, phase 2 trial. Lancet 397, 2060–2069. 10.1016/s0140-6736(21)00520-1 34015342

[B59] Rosenbaum D. Millon A. Fayad Z. A. (2012). Molecular imaging in atherosclerosis: Fdg PET. Curr. Atheroscler. Rep. 14, 429–437. 10.1007/s11883-012-0264-x 22872371 PMC3576137

[B60] Roth G. A. Mensah G. A. Fuster V. (2020). The global burden of cardiovascular diseases and risks: A compass for global action. J. Am. Coll. Cardiol. 76, 2980–2981. 10.1016/j.jacc.2020.11.021 33309174

[B96] Rykaczewska U. Zhao G. A. Saliba-gustafsson P. Lengquist M. Kronqvist M. Bergman P. (2022). Plaque evaluation by ultrasound and transcriptomics reveals BCLAF1 as a regulator of smooth muscle cell lipid transdifferentiation in atherosclerosis. Arterioscler. Thromb. Vasc. Biol. 42, 659–676. 10.1016/j.jacc.2020.11.021 35321563

[B61] Sammartino A. M. Falco R. Drera A. Dondi F. Bellini P. Bertagna F. (2023). Vascular inflammation and cardiovascular disease: Review about the role of PET imaging. Int. J. Cardiovasc Imaging 39, 433–440. 10.1007/s10554-022-02730-9 36255543 PMC9870832

[B62] Schipper H. S. de Ferranti S. (2022). Atherosclerotic cardiovascular risk as an emerging priority in pediatrics. Pediatrics 150, e2022057956. 10.1542/peds.2022-057956 36217888

[B63] Schneider J. W. (2004). Mapping scientific frontiers: The quest for knowledge visualization. J. Am. Soc. Inf. Sci. Technol. 55, 363–365. 10.1002/asi.10383

[B64] Senders M. L. Calcagno C. Tawakol A. Nahrendorf M. Mulder W. J. M. Fayad Z. A. (2022). PET/MR imaging of inflammation in atherosclerosis. Nat. Biomed. Eng. 7, 202–220. 10.1038/s41551-022-00970-7 36522465

[B65] Senders M. L. Que X. Cho Y. S. Yeang C. Groenen H. Fay F. (2018). PET/MR imaging of malondialdehyde-acetaldehyde epitopes with a human antibody detects clinically relevant atherothrombosis. J. Am. Coll. Cardiol. 71, 321–335. 10.1016/j.jacc.2017.11.036 29348025 PMC5995462

[B66] Shen Y. Huang L. Wu X. (2022). Visualization analysis on the research topic and hotspot of online learning by using CiteSpace-Based on the Web of Science core collection (2004-2022). Front. Psychol. 13, 1059858. 10.3389/fpsyg.2022.1059858 36619019 PMC9810495

[B67] Shentu W. Ozawa K. Nguyen T. A. Wu M. D. Packwood W. Xie A. (2021). Echocardiographic molecular imaging of the effect of anticytokine therapy for atherosclerosis. J. Am. Soc. Echocardiogr. 34, 433–442 e3. 10.1016/j.echo.2020.11.012 33253812 PMC8026579

[B68] Singh S. B. Ng S. J. Lau H. C. Khanal K. Bhattarai S. Paudyal P. (2023). Emerging PET tracers in cardiac molecular imaging. Cardiol. Ther. 12, 85–99. 10.1007/s40119-022-00295-1 36593382 PMC9986170

[B69] Song J. W. Nam H. S. Ahn J. W. Park H. S. Kang D. O. Kim H. J. (2021). Macrophage targeted theranostic strategy for accurate detection and rapid stabilization of the inflamed high-risk plaque. Theranostics 11, 8874–8893. 10.7150/thno.59759 34522216 PMC8419038

[B70] Stone P. H. Libby P. Boden W. E. (2022). Fundamental pathobiology of coronary atherosclerosis and clinical implications for chronic ischemic heart disease management-the plaque hypothesis: A narrative review. JAMA Cardiol. 8, 192. 10.1001/jamacardio.2022.3926 PMC11016334 36515941

[B71] Strauss H. W. Narula J. (2017). Imaging vulnerable plaque: A medical necessity or a scientific curiosity? J. Am. Coll. Cardiol. 69, 1792–1794. 10.1016/j.jacc.2017.03.005 28385307

[B72] Synnestvedt M. B. Chen C. Holmes J. H. (2005). CiteSpace II: Visualization and knowledge discovery in bibliographic databases. AMIA Annu. Symp. Proc. 2005, 724–728. 16779135 PMC1560567

[B73] Tu Y. Ma X. Chen H. Fan Y. Jiang L. Zhang R. (2022). Molecular imaging of matrix metalloproteinase-2 in atherosclerosis using a smart multifunctional PET/MRI nanoparticle. Int. J. Nanomedicine 17, 6773–6789. 10.2147/ijn.s385679 36600879 PMC9805955

[B74] Vaduganathan M. Mensah G. A. Turco J. V. Fuster V. Roth G. A. (2022). The global burden of cardiovascular diseases and risk: A compass for future health. J. Am. Coll. Cardiol. 80, 2361–2371. 10.1016/j.jacc.2022.11.005 36368511

[B75] van Eck N. J. Waltman L. (2017). Citation-based clustering of publications using CitNetExplorer and VOSviewer. Scientometrics 111, 1053–1070. 10.1007/s11192-017-2300-7 28490825 PMC5400793

[B76] van Eck N. J. Waltman L. (2010). Software survey: VOSviewer, a computer program for bibliometric mapping. Scientometrics 84, 523–538. 10.1007/s11192-009-0146-3 20585380 PMC2883932

[B78] Vittori A. Cascella M. Leonardi M. Monaco F. Nocerino D. Cuomo A. (2022). VOSviewer-based bibliometric network analysis for evaluating research on juvenile primary fibromyalgia syndrome (JPFS). Child. (Basel) 9, 637. 10.3390/children9050637 PMC9139718 35626815

[B79] Wang D. Yao Y. Wang S. Zhang H. He Z. X. (2021). The availability of the α7-nicotinic acetylcholine receptor in early identification of vulnerable atherosclerotic plaques: A study using a novel ^18^F-label radioligand PET. Front. Bioeng. Biotechnol. 9, 640037. 10.3389/fbioe.2021.640037 33777911 PMC7994753

[B81] Wiegers E. J. A. Mulder M. Jansen I. G. H. Venema E. Compagne K. C. J. Berkhemer O. A. (2020). Clinical and imaging determinants of collateral status in patients with acute ischemic stroke in MR CLEAN trial and registry. Stroke 51, 1493–1502. 10.1161/strokeaha.119.027483 32279619

[B82] Williams A. M. Shah N. P. Rosengart T. Povsic T. J. Williams A. R. (2022). Emerging role of positron emission tomography (PET) imaging in cardiac surgery. J. Card. Surg. 37, 4158–4164. 10.1111/jocs.16992 36345705

[B83] Winter P. M. Morawski A. M. Caruthers S. D. Fuhrhop R. W. Zhang H. Williams T. A. (2003). Molecular imaging of angiogenesis in early-stage atherosclerosis with α_v_ β_3_-Integrin–Targeted nanoparticles. Circulation 108, 2270–2274. 10.1161/01.cir.0000093185.16083.95 14557370

[B84] Wurster T. H. Landmesser U. Abdelwahed Y. S. Skurk C. Morguet A. Leistner D. M. (2022). Simultaneous [18F]fluoride and gadobutrol enhanced coronary positron emission tomography/magnetic resonance imaging for *in vivo* plaque characterization( ). Eur. Heart Journal-Cardiovascular Imaging 23, 1391–1398. 10.1093/ehjci/jeab276 35015852

[B85] Xie Z. Yang Y. He Y. Shu C. Chen D. Zhang J. (2020). *In vivo* assessment of inflammation in carotid atherosclerosis by noninvasive photoacoustic imaging. Theranostics 10, 4694–4704. 10.7150/thno.41211 32292523 PMC7150488

[B86] Xu H. She P. Y. Ma B. X. Zhao Z. Y. Li G. C. Wang Y. B. (2022). ROS responsive nanoparticles loaded with lipid-specific AIEgen for atherosclerosis-targeted diagnosis and bifunctional therapy. Biomaterials 288, 121734. 10.1016/j.biomaterials.2022.121734 35999079

[B87] Yang K. Hu Y. Qi H. (2022a). Digital health literacy: Bibliometric analysis. J. Med. Internet Res. 24, e35816. 10.2196/35816 35793141 PMC9301558

[B88] Yang Y. Luo D. Inam M. Hu J. Zhou Y. Xu C. (2022b). A scientometrics study of the nanomedicines assisted in respiratory diseases. Front. Bioeng. Biotechnol. 10, 1053653. 10.3389/fbioe.2022.1053653 36532565 PMC9757136

[B90] Yuan C. Kerwin W. S. Ferguson M. S. Polissar N. Zhang S. Cai J. (2002). Contrast-enhanced high resolution MRI for atherosclerotic carotid artery tissue characterization. J. Magn. Reson Imaging 15, 62–67. 10.1002/jmri.10030 11793458

[B91] Yuan X. Chang C. Chen X. Li K. (2021). Emerging trends and focus of human gastrointestinal microbiome research from 2010-2021: A visualized study. J. Transl. Med. 19, 327. 10.1186/s12967-021-03009-8 34332587 PMC8325541

[B92] Zhao J. F. Zou F. L. Zhu J. F. Huang C. Bu F. Q. Zhu Z. M. (2022). Nano-drug delivery system for pancreatic cancer: A visualization and bibliometric analysis. Front. Pharmacol. 13, 1025618. 10.3389/fphar.2022.1025618 36330100 PMC9622975

[B93] Zhou Y. Mo M. Luo D. Yang Y. Hu J. Ye C. (2022). Evolutionary trend analysis of research on 5-ALA delivery and theranostic applications based on a scientometrics study. Pharmaceutics 14, 1477. 10.3390/pharmaceutics14071477 35890373 PMC9320574

